# Interference of LPS *H. pylori* with IL-33-Driven Regeneration of *Caviae porcellus* Primary Gastric Epithelial Cells and Fibroblasts

**DOI:** 10.3390/cells10061385

**Published:** 2021-06-04

**Authors:** Weronika Gonciarz, Agnieszka Krupa, Anthony P. Moran, Agata Tomaszewska, Magdalena Chmiela

**Affiliations:** 1 Department of Immunology and Infectious Biology, Faculty of Biology and Environmental Protection, Institute of Microbiology, Biotechnology and Immunology, University of Lodz, Banacha Str. 12/16, 90-237 Lodz, Poland; weronika.gonciarz@biol.uni.lodz.pl (W.G.); agnieszka.krupa@biol.uni.lodz.pl (A.K.); agata.tomaszewska@edu.uni.lodz.pl (A.T.); 2 Department of Microbiology, School of Natural Sciences, National University of Ireland Galway, SW4 Galway, Ireland; Anthony.Moran@nuigalway.ie; 3 Lodz Institutes of the Polish Academy of Sciences, The Bio-Med-Chem Doctoral School, University of Lodz, Banacha Str. 12/16, 90-237 Lodz, Poland

**Keywords:** *Helicobacter pylori*, LPS, IL-33, collagen I, ST2, regeneration

## Abstract

Background: Lipopolysaccharide (LPS) of *Helicobacter pylori* (Hp) bacteria causes disintegration of gastric tissue cells in vitro. It has been suggested that interleukin (IL)-33 is involved in healing gastric injury. Aim: To elucidate whether Hp LPS affects regeneration of gastric barrier initiated by IL-33. Methods: Primary gastric epithelial cells or fibroblasts from *Caviae porcellus* were transfected with siRNA IL-33. Such cells, not exposed or treated with LPS Hp, were sub-cultured in the medium with or without exogenous IL-33. Then cell migration was assessed in conjunction with oxidative stress and apoptosis, activation of extracellular signal-regulated kinase (Erk), production of collagen I and soluble ST2 (IL-33 decoy). Results: Control cells not treated with LPS Hp migrated in the presence of IL-33. The pro-regenerative activity of IL-33 was related to stimulation of cells to collagen I production. Wound healing by cells exposed to LPS Hp was inhibited even in the presence of IL-33. This could be due to increased oxidative stress and apoptosis in conjunction with Erk activation, sST2 elevation and modulation of collagen I production. Conclusions: The recovery of gastric barrier cells during Hp infection potentially can be affected due to downregulation of pro-regenerative activity of IL-33 by LPS Hp.

## 1. Introduction

*Helicobacter pylori* is a Gram-negative pathogenic bacterium colonizing gastric mucosa in humans (the average global infection rate is 50%), which was described by Warren and Marshall in 1983 [1]. Ten to fifteen percent of *H. pylori*-infected individuals will develop chronic inflammatory response, which increases the risk of gastric and duodenal ulcers, stomach cancer and mucosa associated lymphoid tissue (MALT) lymphoma [2,3,4]. Damage of gastric barrier in conjunction with a strong vascularization of the gastrointestinal mucosa facilitates the interaction of *H. pylori* components with fibroblasts, immunocompetent cells, and endothelial cells [5,6]. Major secreted *H. pylori* proteins such as urease converting urea to ammonia, gamma-glutamyl transpeptidase (GGT), neutrophil-activating factor (HP-NAP), vacuolating cytotoxin A (VacA), cytotoxin-associated gene A (Cag A) protein, high-temperature requirement A (HtrA) protein, and other proteins are pivotal for pathogenesis [7,8,9,10,11]. Among cell-bound components, *H. pylori* LPS is involved in gastric tissue destruction and development of inflammatory response. This unique LPS possesses lipid A, which is less acylated and phosphorylated, and due to this, is less endotoxic than typical LPS of Gram-negative rods [12,13,14,15]. It possesses immunomodulatory properties towards macrophages, NK (natural killer) cells and T lymphocytes, whose activity is downregulated [16,17,18,19,20,21,22]. *H. pylori* LPS might be a TLR (Toll-like receptor)-2 agonist, contributing to the NFκB (transcription nuclear factor kappa B) activation independently of a classic LPS receptor TLR4 [13]. Additionally, Lewis x (Le x) and Lewis y (Le y) determinants present in *H. pylori* LPS may be involved in cell signaling due to interaction with DC-Sign (Dendritic Cell-Specific Intercellular adhesion molecule-3-Grabbing Non-integrin)-like receptors [23]. The *H. pylori* invasion and gastric barrier disintegration depend on the local bacterial inoculum and the concentration of soluble bacterial components [21,24]. 

Tissue injury generates secretion of emergency molecules, DAMPs (Damage Associated Molecular Patterns), which alert the innate immune system [25]. Many DAMPs stimulate PRR (pathogen recognition receptors) such as TLRs and cytoplasmic NLRs (Nod-like receptors) or other cell membrane receptors [26]. IL-33 is a member of the IL-1 family of cytokines, which belongs to DAMPs [27]. It drives the immune system to repair tissue injury and eradicate the infection by inducing inflammation and the immune response [28,29,30]. Enhanced expression of IL-33 has been shown in gastric mucosal biopsies from patients positive for *H. pylori*, and higher concentrations of this cytokine are produced in the acute compared to the chronic phase of the infection [31]. Our study on *Caviae porcellus* model experimentally infected with *H. pylori* showed that concentration of IL-33 increased during infection both locally in the gastric tissue and systemically [32]. 

The biological effects of IL-33 depend on its interaction with the ST2 (interleukin 1 receptor-like 1) receptor, which occurs as membranous ligand ST2L or secreted ST2 (sST2), which can serve as a decoy receptor. IL-33 by binding to ST2L activates MAPK (mitogen-activated protein kinases)/Erk (extracellular signal-regulated kinases) signaling and induces various cell activities [33,34,35,36].

Despite its pivotal role as an alarm molecule, IL-33 promotes cell migration and proliferation, and due to this, tissue regeneration in response to injury [37]. However, increased IL-33/ST2 signaling may play a role in the pathogenesis of fibrosis, airway inflammation, allergy, rheumatoid arthritis, collagen vascular diseases or sepsis, and even tumors [35,36,38,39,40]. Furthermore, sST2 may enhance deleterious oxidative stress [34,41]. Various effects moderated by IL-33 seem to be important during *H. pylori* infection, which induces gastric epithelial cell injury and chronic inflammation. Deleterious effects induced by *H. pylori* require activation of regeneration processes to restore local homeostasis.

Previously, we showed in *Caviae porcellus* infected with *H. pylori*, but not control animals, the elevation of oxidative stress and apoptosis in the gastric tissue [42]. These effects were also confirmed in primary gastric epithelial cells and fibroblasts derived from guinea pigs after cell exposure in vitro to *H. pylori* LPS or antigenic compounds (mainly proteins) of the glycine acid extract (GE) from these bacteria. Elevated oxidative stress and apoptosis correlated with increased disintegration of cell monolayers [42]. In response to GE, cells responded with the secretion of IL-33, which stimulated cell migration and proliferation and downregulated activation of Erk-dependent signaling pathway. In contrast, regeneration of cells exposed to LPS *H. pylori* was impaired, possibly due to the lack or inactivation of IL-33 [32]. The mechanism of interference of LPS with IL-33 signaling and an impairment of cell regeneration requires further elucidation. It has been shown that IL-33 and soluble ST2 can activate synthesis of collagens, which may bind kinases receptors, and due to this modulate cell growth and migration [41,43,44]. Cytoplasmic domains of these receptors contain sequences responsible for both positive and negative receptor regulation [43,45].

To explain the role of IL-33 as a pro-regenerative molecule in the gastric barrier and to answer how LPS *H. pylori* modulates the effects driven by IL-33, in this study we used two cell models: primary gastric epithelial cells and fibroblasts of *Caviae porcellus*, which were transfected with the siRNA-IL-33. Then cells were propagated in the culture medium alone or in medium supplemented with exogenous IL-33 and/or LPS *H. pylori*. The following cellular biomarkers were selected to explain how LPS *H. pylori* can modulate the process of IL-33-dependent cell regeneration: lipid oxidation marker—4HNE (4-hydroxy-2-nonenal), Erk activation/phosphorylation (pErk), cell apoptosis, the level of anti-apoptotic Bcl-xL (B-cell lymphoma-extra-large) protein, cell migration, collagen I secretion, and the level of soluble ST2 and IL-33 released by cells to the culture media. 

## 2. Materials and Methods

### 2.1. Cell Cultures

Guinea pig primary gastric epithelial cells were isolated from animals, the usage of which was approved by the Local Ethics Committee (LKE9) for Animal Experiments of the Medical University of Lodz, Poland, which was established by the Ministry of Science and Higher Education in Poland (Decision 44/LB105/2018), and carried out as previously described [32,42]. Briefly, cells were cultured under standard conditions (37 °C, 5% CO_2_) in a mixture of DMEM and Hamʹs F-12 media (ratio 1:1; Sigma-Aldrich, Saint Louis, MI, USA), supplemented with 10% fetal calf serum (FCS), 1% (*N*-2-hydroxyethylpiperazine-*N*-2-ethane sulfonic acid) (HEPES), penicillin (100 U/mL), streptomycin (100 μg/mL), amphotericin B (0.025 μg/mL), L-glutamine (2 mM/mL), epidermal growth factor (Sigma-Aldrich, Saint Louis, MI, USA) 0.01 μg/mL, and 0.005% dexamethasone. 

The guinea pig fibroblasts (CRL-1405) were obtained from the American Type Culture Collection (ATCC, Rockville, Manassas, VA, USA). Cells were routinely grown as a monolayer in complete RPMI-1640 medium (cRPMI) (Sigma St. Louis, MI, USA), at 37 °C in a humidified atmosphere containing 5% CO_2_. Every 2–3 days, the medium was changed, and cells were passaged at 80–90% confluence. The study cells were transfected with siRNA IL-33 and then propagated in the presence or absence of LPS *H. pylori* in the milieu containing or not exogenous IL-33 (ThermoFisher Scientific, Waltam, MA, USA).

### 2.2. Bacterial Stimuli

Cells were treated with LPS from the reference *H. pylori* strain 17874 CCUG (Culture Collection University of Gothenburg, Gothenburg, Sweden), at a concentration of 25 ng/mL, which was adopted on the basis of previously performed experiments [32,42]. LPS was obtained by hot phenol–water extraction and purified by proteinase K, DNase and RNase treatment, followed by ultracentrifugation at 100,000× *g*, at 4 °C, for 18 h, as previously described [46,47,48]. 

### 2.3. IL-33 siRNA Silencing 

Cells were cultured on 6-well plates containing glass coverslips at a density of 1 × 10^6^ cells/well in 1 mL RPMI-1640 or F12: DMEM (Sigma-Aldrich, Saint Louis, MI, USA), without antibiotics, supplemented with 10% fetal calf serum (FCS) and cultivated until complete confluence (37 °C, 5%CO_2_). Next, cells were transfected with the IL-33 siRNA (Santa Cruz Biotechnology, Santa Cruz, CA, USA), according to the manufacturer’s protocol, as previously described [32]. The efficiency of transfection (percentage of positively transfected cells) was evaluated using control siRNA conjugated with isothiocyanate fluorescein (FITC), or was confirmed on the basis of IL-33 secretion into cell culture supernatants using enzyme-linked immunosorbent assay (ELISA) (MyBiosource, San Diego, CA, USA), with a sensitivity of 1.0 pg/mL, in accordance with the manufacturer’s protocol. Next, cells were stimulated with LPS *H. pylori* for 24 h in the presence or absence of exogenous IL-33 (1.5 or 3.0 ng/mL), or sub-cultured in medium alone (control). Finally, the level of IL-33 was assessed. 

### 2.4. Lipid Oxidation—4HNE Production 

The level of 4HNE as a lipid oxidation product was evaluated in IL-33 siRNA-transfected guinea pig primary gastric epithelial cells or fibroblasts (all tested variants). Cells were cultured for 24 h on a 96-well plate (Thermo Fisher Scientific, Waltam, MA, USA) at a density of 1 × 10^6^ cells/well (volume 100 μL) in DMEM: F12 or RPMI-1640 medium alone, respectively (37 °C, 5% CO_2_), or stimulated with *H. pylori* LPS (25 ng/mL), in the presence or absence of IL-33 (3.0 ng/mL). Next, cells were fixed with 4% formaldehyde, incubated with primary rabbit polyclonal anti-4HNE antibodies (Bios, Woburn, MA, USA), followed by FITC-conjugated goat anti-rabbit secondary antibodies (Invitrogen Carlsbad, CA, USA), and counterstained with DNA fluorescent dye 2-[4-(Aminoiminomethyl) phenyl]-1*H*-Indole-6-carboximidamide hydrochloride (DAPI), as previously described [42]. The intensity of fluorescence of cellular 4HNE was estimated using a Victor2 reader (Wallak, Oy, Turku, Finland), at the wavelengths for FITC: excitation 495 nm, emission 519 nm. Cells were visualized using a fluorescence microscope (Zeiss, Axio Scope, A1) at the wavelengths for FITC of excitation 495 nm, emission 519 nm, and for DAPI of 358 nm excitation, 461 nm emission, and at different magnifications (specified in each figure). Five independent experiments were carried out in triplicate for each experimental variant.

### 2.5. Erk Activation/Phosphorylation (pErk)

Primary gastric epithelial cells or fibroblasts transfected with IL-33 siRNA were placed on a 96-well culture plate (Thermo Fisher Scientific, Waltam, MA, USA) at a density of 1 × 10^6^ cells/well (volume 100 μL), in DMEM: F12 or RPMI-1640 medium, respectively (37 °C, 5% CO_2_). Then cells were cultured in the medium alone or in the milieu of *H. pylori* LPS (25 ng/mL), also in the presence or absence of IL-33 (3.0 ng/mL). After 3 or 24 h incubation, cells were fixed with 4% formaldehyde and stained with primary rabbit anti-phospho-Erk1/2 (Thr202/Tyr204) antibodies (Cell Signaling Technology, Danvers, MA, USA), followed by incubation with FITC-conjugated chicken anti-rabbit secondary antibodies (Thermo Fisher Scientific, Waltam, MA, USA), as previously described [32]. Five independent experiments were performed in triplicate for each experimental variant. Erk activation was assessed quantitatively based on the green fluorescence intensity that was measured using a multifunctional Victor2 reader at the following wavelengths for FITC: 495 nm (excitation) and 519 nm (emission).

### 2.6. Cell Apoptosis

Apoptosis of IL-33 siRNA-transfected cells in all tested variants was evaluated by terminal deoxynucleotidyl transferase dUTP nick end labeling (TUNEL) assay (Cell Meter™ TUNEL apoptosis assay kit, AAT Bioques, Sunnyvale, USA), according to the manufacturer’s instructions. Briefly, cells unstimulated or stimulated for 24 h with LPS *H. pylori* (25 ng/mL), in the presence or absence of IL-33 (3.0 ng/mL), were fixed with 4% formaldehyde, stained with Tunnelyte^TM^ red dye, and the cell nuclei were counterstained with Hoechst stain (Cell Meter™ TUNEL apoptosis assay kit, AAT Bioques, Sunnyvale, USA), as previously described [32,42]. Apoptosis was assessed quantitatively based on the red fluorescence intensity that was measured using a multifunctional Victor2 reader, at the following wavelengths: 550 nm (excitation) and 590 nm (emission). Cells fixed with 4% formaldehyde from each experimental variant were incubated overnight at 4 °C with primary antibodies towards anti-apoptotic protein Bcl-xL (Cell Signaling Technology, Danvers, MA, USA), and then stained with FITC-conjugated secondary antibodies (Invitrogen Carlsbad, CA, USA), as previously described [32,42]. Fluorescence intensity was measured using a multifunctional Victor2 reader at the following wavelengths for FITC: 495 nm (excitation) and 519 nm (emission). Five independent experiments were carried out in triplicate for each experimental variant.

### 2.7. Cell Migration—Wound Healing Assay

Cell migration was evaluated in vitro by a wound healing assay—scratch assay, as previously described [6,32,42]. Cells were seeded on 6-well plates at a density of 1 × 10^6^ cells/well in 1 mL cRPMI-1640 or cF12: DMEM medium, supplemented with 2% FCS and 1% penicillin and streptomycin (Sigma-Aldrich, Saint Louis, MI, USA), and cultured until complete confluence. The cell monolayers were scratched with a sterile 200 μL pipette tip (designated as time 0 of wound repair), and then incubated with or without *H. pylori* LPS (25 ng/mL), in the presence or absence of exogenous IL-33 (3.0 ng/mL). Wound images were photographed at 0, 24, and 48 h with a digital camera (Nikon P20, Tokyo, Japan). The scratched areas were measured using ImageJ software version 1.48v (National Institute of Health, Bethesda, MD, USA). Wound healing in cell cultures exposed to LPS, in the presence or absence of IL-33, was expressed as a percentage of cells migrating to the wound zone in comparison to that of the untreated cells, as previously described [6,32,42]. Five independent experiments were carried out with three replicates for each experimental variant. 

### 2.8. Collagen I Assay 

Anti-collagen I antibodies (Bioss Antibodies, MA, USA) were diluted in the carbonate buffer (pH 9.6), applied to the wells of 96-well microtiter plate (1:4 000, 100 µL/well), and incubated for 18 h at 4 °C. Next, wells on the plate were emptied, washed three times with phosphate-buffered saline (PBS) containing 0.05% Tween 80 (PBS/Tween, 250 µL/well), blocked with PBS enriched with 1% bovine serum albumin (BSA, 300 µL/well) for 2 h, at room temperature, and washed five times with PBS/Tween as above. Then, cell culture supernatants were applied to the wells (100 µL/well) and incubated for 1 h at 37 °C, followed by incubation with rabbit anti-collagen I polyclonal antibodies horseradish peroxidase (HRP) conjugated (Bioss Antibodies, MA, USA), diluted 1: 2000 in BSA/PBS/Tween (100 µL/well). After 1 h incubation, the plates were washed and treated with the staining solution (1 mg *o*-phenylenediamine (OPD)/mL phosphate-citrate buffer, pH 5.0 with 0.5 µL/mL 30% H_2_O_2_), 100 µL/well. The color reaction was developed within 20 min in the dark and the enzymatic reaction was stopped with 0.5 M citric acid (50 µL/well). The absorbance was measured at 450 nm using a Victor2 multifunctional reader. Collagen I concentration in cell culture supernatants was determined on the basis of a standard curve (range 1.56–100 µg/mL) for the reference collagen I (Sigma Aldrich, Saint Louis, MI, USA). 

### 2.9. ELISA for ST2

The concentration of ST2 was assessed in supernatants of cells incubated for 24 h, in culture medium only or containing *H*. *pylori* LPS (25 ng/mL), by ELISA (Thermo Fisher Scientific, Waltam, MA, USA), as recommended by the manufacturer (assay sensitivity 4.6 pg/mL). Absorbance was estimated using a Victor2 reader, at a wavelength of 450 nm. Five independent experiments were carried out in triplicate for each stimulation variant. 

### 2.10. Statistical Analysis 

All values are expressed as median values with a range. The differences between the tested variables were assessed using Statistica 12 PL software with a nonparametric Mann-Whitney U test or Kruskal-Wallis test. The results were considered statistically significant when *p* < 0.05.

## 3. Results and Discussion 

During *H. pylori* infection, these bacteria do not colonize the gastric epithelium regularly, rather forming clusters. Due to this, the concentration of bacterial components locally can fluctuate during infection. Colonization of gastric mucosa by *H. pylori* results in the development of chronic inflammatory response and gastric tissue damage [49,50,51,52]. According to the “*theory of danger*”, damaged cells deliver signals, which initiate activation of gastric barrier cells and immunocompetent cells to eliminate infectious agent and restore tissue homeostasis [53]. IL-33 is considered an alert molecule at the gastric epithelial level. Various components of *H. pylori* have the potential to act locally on the epithelium and modulate cytokine secretion, including IL-33. In the previous study using the model of experimental *H. pylori* infection in guinea pigs an increased level of IL-33 has been shown both locally in the gastric tissue and systematically [32,42]. Additionally, primary guinea pig-derived gastric epithelial cells and fibroblasts responded with the production of IL-33 in vitro after exposure to glycine acid extract (GE) of *H. pylori*, but not *H. pylori* LPS. Furthermore, Gonciarz et al. showed that cells treated with LPS *H. pylori* were not able to migrate and proliferate, but rather underwent apoptosis. In this milieu, cells pulsed with LPS *H. pylori* were exposed to a higher oxidative stress than GE-treated cells, and potentially due to this underwent irreversible apoptosis [32]. To explain whether LPS *H. pylori* provides conditions that limit the pro-regenerative activity of IL-33, in this study we engaged models of guinea pig-derived gastric epithelial cells and fibroblasts, which were non-transfected or transfected with IL-33 siRNA. First, we estimated the level of spontaneously produced IL-33 by cells cultured in medium alone or in the presence of LPS *H. pylori*. Then, using siRNA IL-33 transfected cells, we performed experiments in which the level of exogenous IL-33 was added to cell cultures with or without LPS *H. pylori*. Furthermore, we used siRNA IL-33 transfected cells, unexposed or exposed to LPS *H. pylori*, also in the milieu of exogenous IL-33, to evaluate the level of 4HNE as oxidative stress marker, as well as cell apoptosis and activation of Erk (the level of pErk). Next, cell migration was assessed together with the production of pro-regenerative collagen I. Finally, the release of sST2 by siRNA IL-33 transfected cells unexposed or exposed to LPS *H. pylori* was determined.

### 3.1. Assessment of Natural IL-33 or Exogenous IL-33 in Cell Cultures Carried Out in the Presence or Absence of LPS of H. pylori

**Figure 1:**
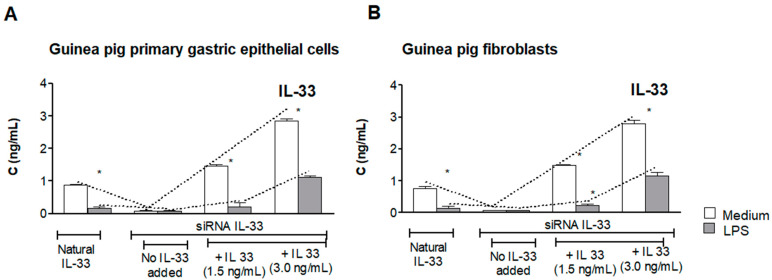
The concentration of IL-33 in cell culture supernatants. The level of IL-33 was assessed in cell cultures of guinea pig-derived primary gastric epithelial cells (**A**) or fibroblasts (**B**). Cells not transfected or transfected with siRNA IL-33 were used. Cells were sub-cultured with or without *H. pylori* lipopolysaccharide (LPS), 25 ng/mL, in the presence or absence of exogenous IL-33 (1.5 or 3.0 ng/mL). The concentration of IL-33 was estimated after 24 h in cell culture supernatants by the enzyme-linked immunosorbent assay (ELISA). Results are shown as median with the range of five experiments performed in triplicate for each experimental variant. Statistical analysis was performed using the nonparametric Mann-Whitney U test with significance at *p* < 0.05 * unstimulated vs. LPS *H. pylori* stimulated cells.

Naive guinea pig-derived primary gastric epithelial cells or fibroblasts, not transfected with IL-33 siRNA, when cultured in medium alone, spontaneously produced 1 ng/mL of IL-33, as showed by ELISA, whereas cells exposed to LPS *H. pylori* released only 0.18 ng/mL of IL-33 (Figure 1A,B). Cells transfected with siRNA IL-33 did not produce this cytokine during experimental time schedule. Next, the cell culture supernatants of transfected cells, which were sub-cultured for 24 h with LPS *H. pylori* in the presence of exogenous IL-33 (1.5 ng/mL or 3.0 ng/mL), were used for the assessment of IL-33. Interestingly, the concentration of IL-33 did not change in cell cultures lacking LPS *H. pylori,* whereas in cell cultures carried out in the presence of bacterial LPS, the level of IL-33 was significantly diminished. In particular, in cell cultures containing LPS *H. pylori* and exogenous IL-33, at the concentration 1.5 ng/mL, the level of this cytokine was diminished from initial 1.5 ng/mL to 0.2 ng/mL. In cell cultures supplemented with 3.0 ng/mL IL-33, the concentration of this cytokine was reduced to 1/3 of initial amount. This may suggest that LPS *H. pylori* provides conditions for degradation or neutralization of IL-33. For further experiments IL-33 was used in a concentration of 3.0 ng/mL. 

Previously, we suggested that neutralization or downregulation of activity of IL-33 in cell cultures supplemented with LPS *H. pylori* could be due to elevated oxidative stress in conjunction with upregulation of apoptosis and delivery of caspases, which may potentially inactivate IL-33 [6,32]. Ali et al. showed that caspase 3 could be responsible for proteolytic degradation and inactivation of IL-33 [54]. The immunomodulatory effect of LPS *H. pylori* towards the host cells through cytokines could also be considered as a potential regulatory mechanism [6,55,56]. 

### 3.2. Oxidative Stress Induced in Cell Cultures Exposed to H. pylori LPS

**Figure 2:**
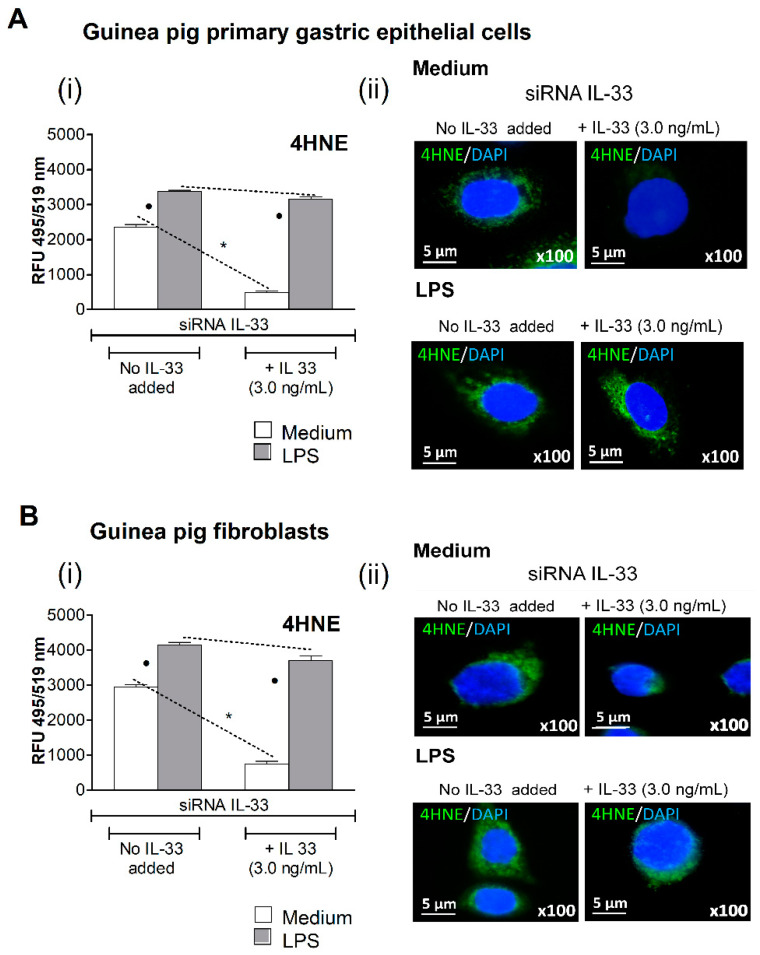
Oxidative stress assessment. The oxidative stress intensity was assessed on the basis of the relative fluorescence of 4-hydroxy-2-nonenal (4HNE), detected by immune staining with anti-4HNE antibodies. Primary gastric epithelial cells (**A**) or fibroblasts (**B**), transfected with siRNA IL-33, not exposed or exposed to *H. pylori* lipopolysaccharide (LPS), 25 ng/mL, in the presence or absence of exogenous IL-33 (3.0 ng/mL), were assessed for 4HNE. (**i**) Fluorescence intensity of 4HNE in gastric epithelial cells or fibroblasts was measured using the ImageJ software version 1.48v (National Institute of Health, United States). (**ii**) Representative images of primary gastric epithelial cells or fibroblasts stained for 4HNE and photographed with a fluorescence microscope (Axio Scope A1, Zeiss, Germany) are shown. Cells were treated with 4′,6-diamidino-2-phenylindole (DAPI), for nuclear staining. Results are shown as median with the range of five experiments performed in triplicate for each experimental variant. Statistical analysis was performed using the nonparametric Mann-Whitney U test with significance at *p* < 0.05. * cells not treated with LPS *H. pylori,* in medium without IL-33 vs. cells not exposed to LPS *H. pylori*, in medium with IL-33; ● cells not treated with LPS *H. pylori* vs. cells treated with LPS *H. pylori*, in medium without or with IL-33.

The oxidative stress in siRNA IL-33 transfected cells grown in medium with or without exogenous IL-33, in the presence or absence of LPS *H. pylori*, was evaluated on the basis of 4HNE, a lipid peroxidation marker. The amount of 4HNE in gastric epithelial cells (Figure 2A) or fibroblasts (Figure 2B) grown in culture medium alone without IL-33 was significantly higher than in cells sub-cultured in medium with IL-33. These results suggested that IL-33 was involved in controlling oxidative stress. By comparison, cells with silenced IL-33 gene treated with LPS *H. pylori* contained more 4HNE than cells in medium alone, which means that oxidative stress was enhanced in response to LPS *H. pylori.* In cell cultures exposed to LPS *H. pylori* in the presence of exogenous IL-33 (Figure 2A,B), the level of 4HNE was not diminished compared to cell cultures pulsed with LPS *H. pylori* in medium lacking IL-33. 

### 3.3. Level of pErk 

**Figure 3:**
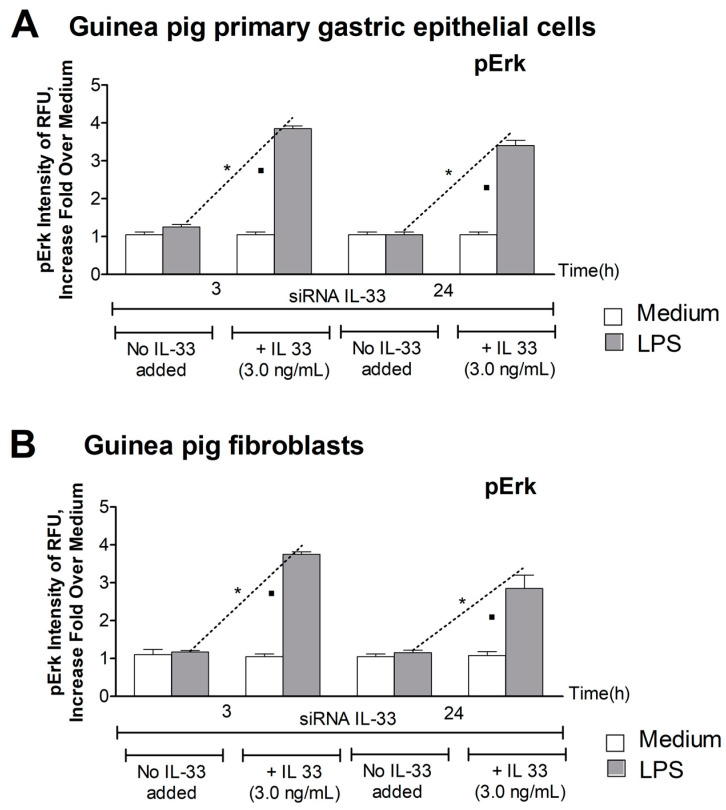
Activation of Erk. The level of activated–phosphorylated Erk (pErk) was estimated in cell cultures of guinea pig primary gastric epithelial cells (**A**) or fibroblasts (**B**), transfected with siRNA IL-33, and then exposed or not exposed to *H. pylori* lipopolysaccharide (LPS), in medium with or without IL-33. Activation of Erk was estimated after 3 h and 24 h on the basis of the relative fluorescence units (RFU) of cells intracellularly stained for pErk with anti-pErk antibodies. Fluorescence was measured in a fluorescence reader (Victor2, Wallac, Oy Turku, Finland) at the following wavelengths: 495 excitation and 519 emission. Results are shown as Fold Over Medium calculated based on fluorescence intensity. Five experiments were performed in triplicate for each experimental variant. Statistical analysis was performed using the nonparametric Mann-Whitney U test with significance at *p* < 0.05. * cells exposed to LPS *H. pylori* in medium without IL-33 vs. cells exposed to LPS *H. pylori* in medium with IL-33; ▪ cells not treated with LPS *H. pylori*, sub-cultured in medium without IL-33 vs. cells treated with LPS *H. pylori*, sub-cultured in medium with IL-33.

The Erk signaling pathway is involved in the positive or negative regulation of different cell activities, including cell growth, differentiation, metabolism, and cell migration, which are initiated in cells in response to various bacterial stimuli or stress conditions [45]. In this study, we estimated the level of phosphorylated Erk (pErk) in siRNA IL-33 transfected cells that were either unexposed or had been exposed to LPS *H. pylori* in medium alone or enriched with exogenous IL-33. The responsiveness of siRNA IL-33 transfected gastric epithelial cells and fibroblasts to LPS *H. pylori* was significantly diminished, so the amount of activated pErk was on the level of medium (Figure 3A,B). However, the subculturing cells with exogenous IL-33 caused an upregulation of cell responsiveness to LPS *H. pylori* to a level approximately four times higher than the medium (Figure 3A,B). In the study, we tested Erk activation after 3 h and 24 h of cell stimulation. The level of phosphorylated Erk in both cell types after 24 h of exposure to LPS *H. pylori* was still high, suggesting the direct involvement of pErk signal protein in the regulation of IL-33- dependent cell activities. 

### 3.4. Influence of LPS H. pylori on IL-33-Driven Downregulation of Cell Apoptosis 

**Figure 4:**
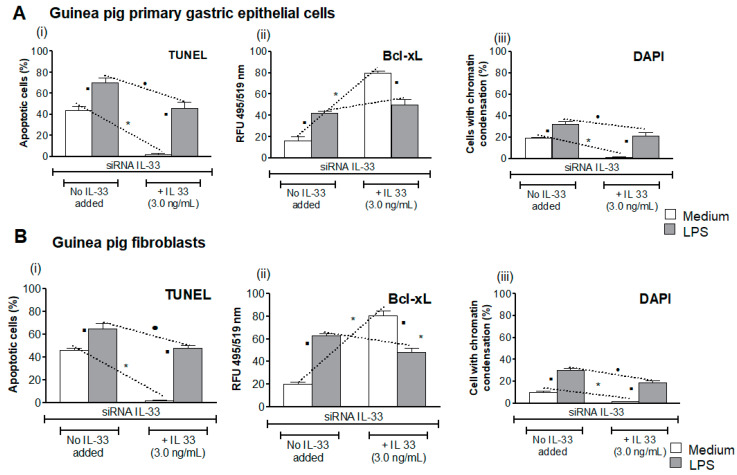
Assessment of apoptosis. Cell apoptosis was estimated by terminal deoxynucleotidyl transferase (TdT)-mediated dUTP nick end labeling (TUNEL) assay (**i**), assessment of expression of anti-apoptotic Bcl-xL protein by using anti-Bcl-xL primary antibodies and secondary antibodies conjugated with isothiocyanate fluorescein (FITC) (**ii**), and 4′,6-diamino-2-phenylindole (DAPI) nuclear staining (**iii**). The results for the guinea pig primary gastric epithelial cells (**A**) or fibroblasts (**B**), transfected with siRNA IL-33, which were not treated or treated with *H. pylori* lipopolysaccharide (LPS), 25 ng/mL, in the culture medium without or with IL-33 (3.0 ng/mL), are presented. The cell fluorescence was measured using a fluorescence reader (Victor2, Wallac, Oy Turku, Finland) at appropriate wavelengths: 495 excitation and 519 emission for FITC, 550 nm excitation and 590 nm emission for TUNEL, and 345 nm excitation and 455 nm emission for DAPI. Results are shown as median with the range of five experiments performed in triplicate for each experimental variant. Statistical analysis was performed using the nonparametric Mann-Whitney U test with significance at *p* < 0.05. *cells in medium without IL-33 vs. cells in medium with IL-33, not treated with LPS *H. pylori*; ● cells treated with LPS *H. pylori*, in medium without IL-33 vs. cells treated with LPS in medium with IL-33; ▪ cells not treated with LPS *H. pylori* vs. cells treated with LPS *H. pylori*, in medium without or with IL-33.

Increased oxidative stress may result in elevation of cell apoptosis, which might potentially be upregulated by metalloproteinase (MMP)-9 [57]. Previously, we showed that experimental infection with *H. pylori* in guinea pigs resulted in increased oxidative stress and apoptosis in gastric epithelial cells in conjunction with upregulation of MMP-9 [32,42]. Here, we showed that approximately 40% of gastric epithelial cells or fibroblasts transfected with siRNA IL-33 sub-cultured in medium alone without IL-33 underwent apoptosis, as shown in the TUNEL assay (Figure 4A,B). The level of cells undergoing apoptosis was significantly diminished in the presence of IL-33, which was added to the culture medium. This may suggest that IL-33 alone can promote the restoration of cell homeostasis, potentially due to a reduction in the level of oxidative stress, the inhibition of Erk activation, and apoptosis. By comparison, 73% and 62% of apoptotic cells were found in cell cultures of gastric epithelial cells and fibroblasts, respectively, after exposure of cells to LPS *H. pylori* in medium without IL-33, which means that apoptosis in these experimental variants was triggered by LPS. In the presence of LPS *H. pylori* and IL-33 in cell cultures, the percentage of apoptotic cells was only slightly reduced (approximately 10%). DAPI staining confirmed trends in the amount of cells undergoing apoptosis (Figure 4A,B). 

Furthermore, in cells not exposed to LPS *H. pylori*, which were grown in medium with IL-33, enhanced expression of anti-apoptotic protein Bcl-xL was shown as compared to cells propagated in medium lacking IL-33. This was in line with the diminished number of apoptotic cells in the TUNEL assay. Importantly, in cells exposed to LPS *H. pylori*, in the presence of IL-33, the intensity of fluorescence of anti-apoptotic Bcl-xL protein was on the similar level (Figure 4A,B). These data indicate that LPS effectively promoted cell apoptosis, and that exogenous IL-33, which was added to the culture medium, did not effectively diminish LPS-driven apoptosis. Increased apoptosis of cells exposed to LPS in the medium with IL-33 was in line with pErk increase.

### 3.5. Influence of LPS H. pylori on IL-33-Driven Cell Migration 

**Figure 5:**
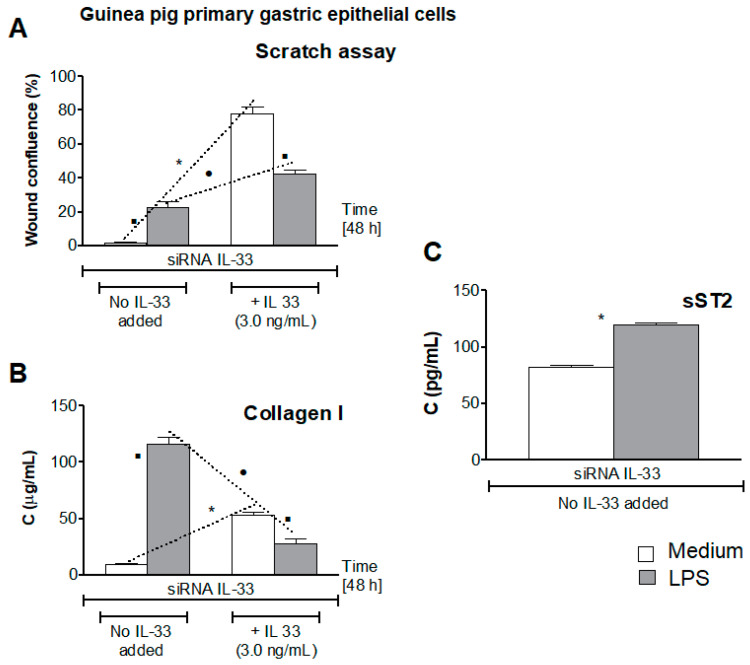
Pro-regenerative activity of gastric epithelial cells. The cells transfected with siRNA IL-33, which were not exposed or exposed to lipopolysaccharide (LPS) *H. pylori* (25 ng/mL), were sub-cultured in medium without or with IL-33 alone 3.0 ng/mL. Cell monolayers were scratched and then the cells’ ability to migrate was assessed (**A**). Cell culture supernatants were used for assessment of collagen I concentration (**B**) by the enzyme-linked immunosorbent assay (ELISA). Furthermore, the level of soluble ST2 (sST2), was assessed by the ELISA in cell cultures of siRNA IL-33 transfected cells not exposed or exposed to LPS *H. pylori* (**C**). Results are shown as median with the range of five experiments performed in triplicate for each experimental variant. Statistical analysis was performed using the nonparametric Mann-Whitney U test with significance at *p* < 0.05. * cells in medium without IL-33 vs. cells in medium with IL-33, not treated with LPS *H. pylori*; ● cells treated with LPS *H. pylori*, in medium without IL-33 vs. cells treated with LPS in medium with IL-33; ▪ cells not treated with LPS *H. pylori* vs. cells treated with LPS *H. pylori*, in medium without or with IL-33.

**Figure 6:**
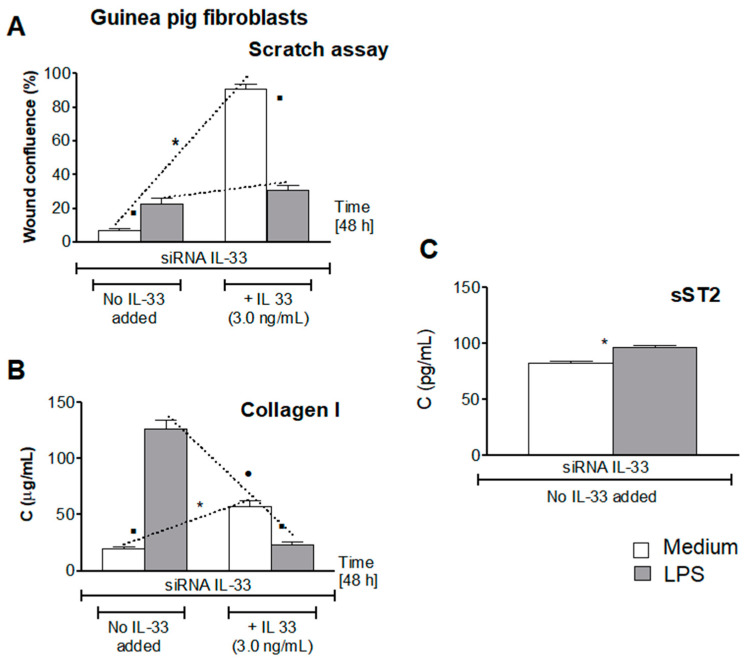
Pro-regenerative activity of guinea pig fibroblasts. Cells transfected with siRNA IL-33, which were not exposed or exposed to lipopolysaccharide (LPS) *H. pylori* in medium without or with IL-33 3.0 ng/mL, were used. Cell monolayers were scratched and then the cells’ ability to migrate was assessed (**A**). Cell culture supernatants were used for assessment of collagen I concentration (**B**) by the enzyme-linked immunosorbent assay (ELISA). Furthermore, the level of soluble ST2 (sST2) was assessed by the ELISA in cell cultures of siRNA IL-33 transfected cells not exposed or exposed to LPS *H. pylori* (**C**). Results are shown as median with the range of five experiments performed in triplicate for each experimental variant. Statistical analysis was performed using the nonparametric Mann-Whitney U test with significance of *p* < 0.05. * cells in medium without IL-33 vs. cells in medium with IL-33, not treated with LPS *H. pylori*; ● cells treated with LPS *H. pylori,* in medium without IL-33 vs. cells treated with LPS in medium with IL-33; ▪ cells not treated with LPS *H. pylori* vs. cells treated with LPS *H. pylori,* in medium without or with IL-33.

In this study, we asked whether LPS *H. pylori* modulates the IL-33-induced cell migration in response to diminished cell integrity due to oxidative stress and cell apoptosis. Collagen I is an important pro-regenerative factor, which promotes cell migration [45]. Recently it was shown that cell growth can be induced by collagen I via Erk receptors, designated as DDR (dictyostelium discodein protein)1 and DDR2, which are present in gastrointestinal epithelial cells [43]. As shown in Figure 5A, primary gastric epithelial cells, transfected with siRNA IL-33, were not able to migrate and minimize the scratch in wound healing assay in the absence of IL-33. However, cells healed the wound effectively, up to 80% confluence, in the milieu of exogenous IL-33. The ability of gastric epithelial cells to migrate in the presence of IL-33 was related to elevated production of collagen I (Figure 5B). Similar effects were demonstrated in wound healing assay with guinea pig fibroblasts sub-cultured in medium without or with IL-33 (Figure 6A,B). 

By comparison, gastric epithelial cells or fibroblasts exposed to LPS *H. pylori* in medium without IL-33 showed 20% of wound confluence. In these conditions, both cell types released over 100 pg/mL of collagen I (Figure 5 and Figure 6). This could be the extremal reaction of cells towards elevated oxidative stress and disintegration of cells due to apoptosis. Interestingly, cells exposed to LPS *H. pylori* in the milieu of IL-33 showed some further increase in cell migration; however, the confluence reached only 40%, as compared to 80% of confluence of cells, which were not treated with LPS *H. pylori* but propagated in medium with IL-33. Concentration of collagen I was on the level of 25 pg/mL in cell cultures with LPS and IL-33 vs. 50 pg/mL in cell cultures without LPS but with IL-33 (Figure 5A,B and Figure 6A,B). These results indicate that IL-33 was involved in cell migration in conjunction with upregulation of collagen I production. On the other hand, high collagen I response of cells exposed to LPS *H. pylori* could reflect the response of cells to enhanced oxidative stress and apoptosis. Hoyt et al., using the model of cultured pulmonary artery endothelial cells, showed that collagen is a survival factor against LPS-induced apoptosis [58]. Recently, Krupa et al. showed the increased collagen I response of vascular endothelial cells exposed to LPS *H. pylori* [59]. In this study, in the milieu of LPS *H. pylori* and IL-33, migration of cells did not exceed 40% confluence, which may suggest some interference between bacterial LPS and host IL-33 signaling. Downregulation of collagen I production in such conditions may potentially limit cell migration. Collagens are sensitive to MMPs degradation [43,45]. We have previously shown that in vitro exposure of gastric epithelial cells or fibroblasts to LPS *H. pylori* was related with elevated production of MMP-9 [32,42]. Additionally, Gonciarz et al. showed that the concentration of MMP-9 in guinea pigs infected with *H. pylori* significantly increased locally in the gastric tissue and systemically [42]. It is possible that MMP-9 delivered by cells responding to LPS *H. pylori* could induce decomposition of collagen I and due to this may slow down cell migration. Degradation of collagen by MMPs was suggested as a potential mechanism of escaping of neoplastic cells because of the loss of their ability to adhere [43,45].

Recently, an emerging role of the IL-33/ST2 axis in gut mucosal tissue repair has been suggested [60,61,62]. IL-33 achieves its function through the ST2 cell membrane receptor. However, soluble ST2 can be released by different cells, including gastrointestinal cells. It was showed that administration of sST2 protected mice against endotoxin-induced shock [63,64], and ischemia/reperfusion-related lethality [65]. This was due to attenuation of TLR-4 expression [63]. Furthermore, Matilla et al. showed that sST2 induced activation of fibroblasts and production of collagen due to upregulation of neurophilin-1 (NRP-1) [66]. Recently, Takenaga et al. showed enhanced growth of tumor cells by sST2 in a murine pancreatic cancer model [67]. By comparison, the study of Alimoto et al. revealed that sST2 inhibited growth of colorectal cancer malignant cells [68]. This may suggest either positive or negative sST2 regulations.

In this study, we showed that guinea pig primary gastric epithelial cells transfected with siRNA-IL-33 released more sST2 (120 pg/mL) when propagated in the culture medium with LPS *H. pylori* than cells grown in medium alone (75 pg/mL), *p* < 0.05 (Figure 5C). Similarly, guinea pig fibroblasts transfected with siRNA-IL-33 sub-cultured in culture medium alone produced less sST2 (75 pg/mL) than cells pulsed with LPS *H. pylori* (100 pg/mL), *p* < 0.05 (Figure 6C). It is worth mentioning that serum levels of sST2 were also significantly increased in guinea pigs infected with *H. pylori* (61 ± 8.7 pg/mL) vs. uninfected animals (9.4 ± 2.3 pg/mL), which was assessed by using guinea pig serum samples from the previous study [32,42]. Matilla et al. showed that in cell cultures exposed to sST2, peroxide production and secretion of pro-inflammatory cytokines IL-6 and IL-1β increased in conjunction with upregulation of collagen I production [34,66]. It is possible that in this study an increased level of sST2 released by cells in response to LPS *H. pylori* could drive the production of collagen I, and thus initial cell migration, in the absence of IL-33. However, Martinez-Martinez et al. showed that sST2 increased proteolytic activity of MMP-9 [40]. It is known that MMPs are the main extracellular matrix enzymes causing collagen degradation [69]. It is possible that in our experimental model LPS, *H. pylori* induced the cascade of events, which resulted in an increased production of sST2 and MMP-9, the proteolytic activity of which was enhanced. It is possible that the activity of IL-33 can be downregulated in such a milieu. It is also possible that sST2, released extensively by cells exposed to LPS *H. pylori* as IL-33 decoy, may neutralize this cytokine and lower its pro-regenerative activity. Due to sST2/IL-33 complexing the collagen production and cell migration can be diminished in the milieu of IL-33 and LPS *H. pylori.* However, IL-33 may provide optimal conditions for cell regeneration due to balancing the overproduction of collagen I, which can be driven in the presence of LPS *H. pylori* and sST2. It was shown that overproduction of collagen in response to injury induced by LPS could promote the development of fibrosis [41].

In summary, infection with *H. pylori* and the components of these bacteria, including LPS, can cause damage to the gastric epithelium and fibroblasts. This requires the initiation of repair processes to restore homeostasis. Wound healing is a complex process, including inflammation, cell migration and proliferation, wound contraction, and collagen metabolism. It encompasses complex interaction between cells, and with the extracellular matrix, as well as cytokines [70]. It was shown on a model of skin wound healing that bacterial endotoxin in low concentration can have no effect or be beneficial in regeneration process, whereas in excessive concentrations it can cause inhibition of cell proliferation, and destruction of cellular components and extracellular matrix through its decomposition. Due to this, slow wound healing and an even deepening and expansion of the wound can occur [71,72,73]. Our study showed that LPS *H. pylori* may initiate deleterious effects towards gastric barrier cells due to increasing oxidative stress and apoptosis in conjunction with elevated production of caspases, MMP-9 and sST2. In response to cell injury, in the milieu of LPS *H. pylori*, cells responded through the acceleration of collagen I, which could be beneficial in an initial stage of the healing process. However, the conditions provided by LPS *H. pylori* might in fact slow down the regeneration process, potentially due to the interference from the pro-regenerative activity of IL-33. It would be interesting to ascertain whether the secretion of different cytokines in the cell culture, in the presence of LPS *H. pylori,* dictates the conditions regulating wound healing, and whether TLR-4 expression can provide an explanation for our observations. Previously, Duncan et al. showed that LPS-driven interferons were responsible for the inhibition of collagen production by fibroblasts and their proliferation [74]. Chakravortty et al., on a model of mast cells, showed that there was a potential cross talk between LPS/TLR-4 and IL-33/ST2 signaling through Myd 88. Furthermore, they showed that LPS slightly upregulated expression of ST2 [75]. In this study, the level of sST2 was higher in siRNA IL-33 transfected gastric epithelial cells or fibroblasts exposed to LPS *H. pylori* than in cells not treated with LPS. It might be possible that in the absence of IL-33, ST2 could upregulate LPS/TLR signaling resulting with increased collagen I production and cell migration. 

Ball et al. suggested that IL-33 can engage additional receptors such as SIGIRR (Single Ig IL-1-related receptor), which negatively regulates TLR-mediated responses [76]. Potentially, even low levels of IL-33 can induce hyporesponsiveness of cells to LPS, and provide a homeostatic mechanism for limiting the negative effects driven by this bacterial component, possibly due to high acceleration of collagen I. In our study, gastric epithelial cells or fibroblasts exposed to LPS *H. pylori* produced very high levels of collagen I, which stimulated cell migration. However, in the milieu of IL-33, the amount of collagen I was diminished, which was followed by limited cell migration. This effect may potentially be protective against collagen-dependent fibrosis. However, downregulation of cell migration in the presence of LPS *H. pylori* and IL-33 may slow down gastric tissue regeneration and promote the maintenance of inflammation. *H. pylori* LPS-driven disintegration of the gastric barrier can favor the interaction of *H. pylori* components with the vascular endothelium and immunocompetent cells. The microvascular endothelium has been suggested as a key target during gastric mucosal injury [77]. Such a cascade of events may potentially link local *H. pylori* infection in the gastric mucosa with the development of systemic diseases. 

## 4. Conclusions

Our study showed that LPS *H. pylori* may initiate deleterious effects towards gastric barrier cells due to increasing oxidative stress and apoptosis in conjunction with elevated production of caspases and sST2. In response to cell injury, in the milieu of LPS *H. pylori*, primary gastric epithelial cells and fibroblasts, derived from stomach tissue of *Caviae porcellus*, responded by the acceleration of collagen I, which could be beneficial in an initial stage of the healing process. However, the conditions provided by LPS *H. pylori* might slow down the repair process of gastric tissue cells, potentially due to interference with the pro-regenerative activity of IL-33, which is expressed by enhancing cell migration. Slowing down the regeneration of the gastric barrier in the milieu of LPS *H. pylori*, due to neutralization of IL-33-induced effects, may contribute to the maintenance of inflammation, locally as well as systemically.

## Data Availability

Data are contained within the article or supplementary materials.
